# Saint George's Hospital

**Published:** 1829-01-01

**Authors:** 


### VIII.

### ST. GEORGE'S HOSPITAL.

I. Cancer of the Tongue—apparently bad, if not fatal, Effects of the Exhibition of a small Quantity of Arsenic.

This case is important in a practical view. Benjamin Collins, 63 years of age, a smith by occupation, was received into St. George's, on the 27th of August, under the care of Mr. Brodie. He stated that he had always been accustomed to smoking, and noticed, about Christmas last, a small sore spot on the left side of the tongue. The organ was scarcely if at all enlarged, and the inconvenience so slight that a month or two elapsed before he applied for advice. The soreness was but trifling, and rather consisted in a sense of smarting than actual pain, but the disease advanced unchecked and unmitigated by art to the time of his admission. A month or rather more before this period, the glands on the left side of the neck began to swell, and one of them shortly ulcerated, whilst those on the opposite side were several times enlarged, but as often subsided. For some time he had been totally unable to swallow any solid food. Such is the history; let us now turn our attention to the symptoms which were actually presented.

The tongue was firmly fixed, at least its left half was so, the right, for a very narrow strip, being free and unembarrassed. The whole of the organ was inclosed within the boundary formed by the teeth, beyond which it could not be protruded, its left side having likewise received the impressions of the molar and bicuspids. On the dorsum of the tongue, which was greatly hardened, a prominence was seen about the size of a nut, to the left of which was a hollow large enough to receive the extremity of the thumb, formed by ulceration, and tender to the touch. Little pain was complained of; the discharge of saliva was constant and profuse; the teeth in a very bad state; the alveoli sound. The glands on the left side of the neck, beneath the jaw, were enlarged, and one or more in a state of ulceration—those on the right side much enlarged also. The general appearance was sallow and unhealthy—the bowels inclined to be costive—the flow of saliva prevented much sleep—the appetite was not much impaired, but nothing save liquids could be swallowed. The whole case presented a hopeless and pitiable picture of scirrhus of the tongue.

As diseases of the tongue occasionally yield to the powers of arsenic, at any rate as that remedy was as likely to do good as any other, Mr. Brodie directed five drops of the liquor arsenicalis to be taken thrice daily, with a drachm of mucilage of acacia, and an ounce and a half of distilled water. At the end of three days, the quantity of the liquor arsenicalis was raised from five drops to seven; but, Sept. 5th, eight days from commencing the treatment, vomiting being produced by the medicine, the latter was omitted. We saw him on the 8th, and he looked very ill indeed; the countenance was anxious and distressed—he complained of little sickness, but a dull aching pain in the back of the head—the pulse was quick and bounding—the bowels confined—sleep and appetite destroyed—discharge of saliva more profuse than before, and a little tinged with blood. The symptoms assumed a worse aspect every day, the saliva continued flowing in amazing quantities, and the patient expired on the evening of the 11th.

*Dissection.* The left side of the tongue was almost entirely eaten away by ulceration, the cavity being foul, and its edges everted and black. On making a section of the right side, a scirrhous tubercle was found imbedded in its substance, though outwardly this part might have seemed to have been sound. The parts in the neighbourhood were inflamed, in one spot the soft palate had even proceeded to ulceration. On making an incision in front of the trachea, and dissecting the parts there, the glandulæ concatenatæ were more or less indurated, enlarged, or even ulcerated. The pylorus was thicker than it should be, but little, if at all condensed or hardened. The mucous membrane of the stomach was a little inflamed, and its rugæ were blackened. The peritoneum investing the spleen was in parts cartilaginous, the appearance being happily compared, by Dr. Hewett, to portions of tallow dropped and congealing on the membrane. The mesenteric glands were indurated and enlarged, the rest of the viscera were healthy.

Mr. Brodie remarked in the dead-house, and the same must strike all who reflect on the case, and attentively regard the appearances displayed on dissection, that the death of the patient would appear to be immediately owing to the remedy rather than the original disease. It is true that the quantity taken was small, little more than a grain of the arsenic in substance,* but then, it should be remembered, that the patient was prevented from taking solid nourishment, and labouring under spontaneous salivation at the time;† circumstances calculated to favour absorption. His appearance, a few days before his death, was exactly

---

* The patient took 150 minims of the liquor arsenicalis in the space of eight days.

† It is known that salivation is not an unfrequent consequence of poisoning by arsenic. In this case, the salivation which previously existed was certainly increased.

that of a person whose system is affected by the poison of mercury or other pernicious drug; so wan, so cadaverous, that the beholder is involuntarily struck with it. Perhaps it was fortunate that the arsenic had this effect, for it saved him from weeks or even months of disgusting and irremediable misery.

### II. CASE OF CUT-THROAT—SYMPTOMS, APPARENTLY, OF DISEASE OF THE BRAIN.

William Fry, fifty-two years of age, was brought to St. George's on the 30th June, having just cut his throat with a common dinner knife. The wound was extensive; nearly two-thirds of the trachea were divided between its first ring and the cricoid cartilage; whilst the bleeding was so free, as to require three vessels to be tied. A stitch being passed between the trachea and cricoid cartilage, the wound of the windpipe was effectually closed, the ligature causing but little irritation, and enabling the patient to breathe through the mouth. The external wound was united by adhesive straps only. The man was of a lymphatic habit of body, with a heavy and suicidal aspect; had met with reverses; and been for some time in a low desponding state, attended with symptoms which induced the practitioner who saw him to order his head to be shaved, and the back of the neck to be blistered and cupped.

For the first few days he appeared pretty well, but then it was observed that the pulse intermitted about every third beat, the tongue was dry and red, and the patient complained of much pain in the head. On the 2d of July, the pain in the head was severe, and ten ounces of blood were abstracted from the arm, and salines with antimonial wine, directed to be taken thrice daily. The blood was cupped and buffed, on which account venesection, to the same amount, was practised again upon the 4th. After this, he fell into a lethargic state, insensible to every thing said or done around him; refusing both medicine and food; complaining of, and apparently suffering, no pain; and evincing no further disposition to suicide. The tongue was coated—the eyes glassy, and the pupils very sluggish—the pulse weak, low, and intermitting.

In this condition he remained for a fortnight or more, when he gradually recovered. The pulse became regular—the torpor passed away, and, in the course of a short time, the patient was dismissed, not perfectly free from the symptoms of some obscure affection of the brain, but still so far improved, as to be no longer fit for the inmate of a hospital.

### III. CURIOUS ENLARGEMENT OF THE LEFT CHEEK—ANOMALOUS PAIN IN THE HEAD, AND OTHER SYMPTOMS.

The impenetrable obscurity in which are involved the more chronic affections of the brain, at least as regards their diagnosis, is well known to all who have met with such cases in practice. In females, especially young ones, this obscurum is rendered obscurius, by those nervous and hysterical symptoms which frequently counterfeit serious mischief, and occasionally mask it when present. Nothing is, in fact, more perplexing, than the junction of hysteria with actual disease. We shall detail the particulars of the following case, without giving any opinion on its nature.

Mary Rivers, between 18 and 19 years of age, was admitted into hospital on the 12th of August, and fell to the care of Mr. Brodie.

The right side of the face was sunken and lank, the left preternaturally full, though not from any solid deposition of lymph, nor from œdema. The cheek still retained all its pristine pliancy and softness, though greatly enlarged, and, as it were, hypertrophied. The jaw was firmly closed, and could not be opened by any moderate force, to more than the eighth of an inch, though no disease was found in the brain, the alveoli, or teeth. The head appeared to hang in a trifling degree towards the left, whilst its motions were imperfect, on the whole, and incapable of their usual development. The sterno-mastoidei and muscles of the neck appeared unaffected, the contraction being chiefly in the pterygoid muscles of the jaw. The left side of the head was the seat of continual and distressing pain, and some was experienced on pressing posterior to the ear, as well as before it and below it, immediately in the situation of the portio dura. The sense of hearing was obtuse, on the side affected very much so; the eyes weak and sore upon exertion; the speech low and drawling; the countenance heavy; the girl altogether inanimate and drowsy.

From the statement of the patient, which was clear and satisfactory, the disease had begun with the pain in the head, about twelve months previous to admission, whilst the swelling of the cheek was only of six months' duration. About the time that the swelling appeared, the catamenia, which had hitherto been regular, ceased, and never again re-appeared. Though the appetite was bad, she had not been affected with nausea or vomiting, but suffered at first from a beating and throbbing in the ear. Leeches, blisters, &c. &c. had been freely employed by the surgeon in the part or the country where she was, but never with any relief to her sufferings. The pain, we should mention, was worse during the night, and prevented her obtaining any sleep.

The treatment consisted in first opening the bowels by calomel and black draught, applying a caustic issue behind the ear, and putting her upon a course of two grains of the submuriate of mercury, with half a grain of powered opium twice a day, for upwards of three weeks. The medicine at the end of this time was discontinued, and the subsequent plan has been blistering to the nucha, and the warm-bath at night. The state of the patient is by no means improved, the countenance being even more unfavourable than before, and the pain in the side of the head very bad, though not exactly seated in the former situation.

Mr. Brodie's opinion on the nature of the case is not yet made up, and we give the details in order to shew an ambiguous and anomalous assemblage of symptoms, which occasionally presents itself, and puzzles the practitioner. The affection has certainly many of the characters presented by organic disease of the brain, or the bones which encase it; characters, however, whose distinguishing mark is obscurity. Though maladies essentially hysterical or nervous have many of the features in common with those which depend upon actual disorganization, there is generally a certain expression of countenance, a *je ne sçais quoi*, in the latter, shewing, as it were, that the system is aware of the difference, even though the doctor is not. This peculiar expression, which may perhaps be recognized, but cannot be defined, appears to be present in the subject of the case we have related, and offers a feeble, it may be fallacious, indication.

---

